# Revisiting Clonal Evolution Through the Light of Retrotransposons

**DOI:** 10.1002/bies.70078

**Published:** 2025-10-09

**Authors:** Anaïs Lamoureux, Emilie Elvira‐Matelot, Françoise Porteu, Lucie Laplane

**Affiliations:** ^1^ CNRS, UMR8590 IHPST University Paris 1 Panthéon‐Sorbonne Paris France; ^2^ Université Paris‐Saclay Gustave Roussy, Inserm U1287, Hematopoietic stem cells and the development of myeloid malignancies Villejuif France

**Keywords:** cancer cell, cell fusion, clonal evolution, horizontal gene transfer, lineages, retrotransposons

## Abstract

The clonal evolution model provides a framework for understanding the evolution of cancer cells. According to this model, cancer cells accumulate genetic mutations over time, and these mutations are passed down to their descendants, leading to genetic diversity within the tumor. Some of these mutations confer selective advantages, causing certain lineages of cancer cells (clones) to dominate and expand. However, this model is rooted in certain conceptual assumptions, which we propose to revisit by considering the potential involvement of retrotransposons in cancer initiation and progression. In recent years, it has become evident that transposable elements, particularly retrotransposons, play a significant role in driving cancer transformation and progression. We first review how current knowledge about retrotransposon activity aligns with the clonal evolution model by highlighting its ability to modulate cancer cell fitness. We then take a forward‐looking perspective to explore additional ways retrotransposons may also influence clonal dynamics beyond the current model.

## Graphical abstract

We propose integrating retrotransposons into the clonal evolution model of cancer. Retrotransposons can modulate cancer cell fitness, potentially influencing clonal dynamics. They might also induce lineage violations via cell fusion or horizontal transfer. Recognizing their role could refine the model and suggest novel therapeutic strategies targeting retrotransposon activity.

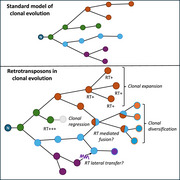

## 1 Introduction

Retrotransposons are repeated sequences of DNA that represent about 42% of the human genome. They have often been thought as junk DNA, an assumption that has since been called into question [1, 2, 3, 4, 5, 6]. They have played a major role in the evolution of mammal and human genomes [7, 8, 9]. Their derepression can participate in cancer progression [10, 11]. In this perspective, we discuss the role that retrotransposons play in the evolution of cancer cells. Currently, the evolution of cancer cells is represented through the clonal evolution model, inspired by population genetics and the modern synthesis. According to this classical view, cancer cells acquire mutations, which are transmitted to their descendants, generating genetic intratumor heterogeneity through space and time [12]. Although most mutations are neutral, some may provide selective advantages leading to the overexpansion of the clone (the population descending from the initial cell that acquired the mutation). Conversely, other mutations may be deleterious, leading to the decrease or disappearance of the clone bearing these mutations. However, reliance on the evolutionary synthesis has been challenged by biologists for being too reductive or simplistic, giving rise to several alternative frameworks currently gathered under the umbrella of the “extended evolutionary synthesis” [13]. Similarly, although the genetic model of clonal evolution has proved useful in oncology, it has started to reach its limits, leading to several attempts at extending the model beyond the genes [14]; for example, by taking into account epigenetic clonal evolution [15], by emphasizing the importance of ecology [16], or by suggesting a multispecies view of clonal evolution accounting for the role of intratumoral bacteria [17], as well as by integrating more evolutionary mechanisms, such as the possibility of neutral evolution [18]. Nonetheless, retrotransposons remain a missing part of this puzzle. It has become clear in recent years that retrotransposons can play an important role in transformation and cancer progression. As such, it is thus time to pause and reflect on how to integrate their involvement in the clonal evolution model.

## 2 Retrotransposons Influence Cancer Cell Fitness

Retrotransposons are a type of mobile genetic element. In contrast with transposons, which use a cut and paste mechanism to transpose in the genome (the transposon literally moves from one place to another), retrotransposons use a copy and paste mechanism, which result in a multiplication of its copies. Retrotransposons are classified into two main types: Long Terminal Repeat (LTR) and Non‐LTR retrotransposons. LTR retrotransposons, or endogenous retroviruses (ERVs), originate from ancient viral infections of germ cells. A fraction of ERVs retain the retrotransposition mechanism characteristic of retroviruses, which involves reverse transcription of RNA into DNA, and integration into the host genome via integrase [7]. Non‐LTR retrotransposons include long interspersed nuclear elements (LINEs) and short interspersed nuclear elements (SINEs). LINEs are autonomous elements encoding reverse transcriptase and endonuclease, enabling their retrotransposition through target‐primed reverse transcription, directly integrating into DNA at the site of insertion. SINEs are non‐autonomous elements that rely on the LINE machinery to propagate. Retrotransposition can impact genomic stability, introduce mutations or change the transcriptome of the cell [19, 20], leading to multiple consequences, both negative (e.g., cell death) and/or positive (e.g., contributing to cell diversification). Retrotransposons that can no longer retrotranspose may still be transcriptionally active and can serve for example as secondary promoter or transcription enhancer. ERVs and LINE‐1 have been implicated in various cancers [21], and are now perceived as potential therapeutic targets [22, 23, 24, 25]. Retrotransposons raise an interesting conundrum for cancer research. In cancer cells, the repression of retrotransposons is removed [9, 26], a phenomenon so widespread that it is often referred to as a hallmark of cancer [27, 28, 29], but this puts the cancer cells’ survival at risk.

### 2.1 Retrotransposons Can Decrease Cancer Cells’ Fitness

Retrotransposons are usually repressed [30], and their derepression in cancer can be deleterious to the cancer cells. First, the reexpression of retrotransposons can lead to adaptive or innate immune responses. Transcription of ERVs can produce neoantigens presented at the cell surface by major histocompatibility complex (MHC), which can induce an adaptive immune response against cancer cells [31]. The presence of retrotransposon derived double‐stranded RNA (dsRNA), double‐stranded DNA, or complementary DNA can be detected as foreign material by cell nucleic acid sensors such as MDA5/RIG1 or cGAS‐STING [32, 33]. This leads to interferon‐type I (IFN‐I) production and induces antiviral immune response, a process called viral mimicry response [34, 35] For example, Liu et al. showed that in a mouse model of colorectal adenocarcinoma, the knockout (KO) of *PHF8*, a histone demethylase that silences retrotransposons, lifts this silencing, leading to induction of viral mimicry response [36]. The process resulted in a growth disadvantage of *PHF8* KO cells over control cells in an in vivo competition assay [36]. It has been shown that hypomethylating agents, used to treat some cancers, relieve the repression of retrotransposons and lead to dsRNA formation, IFN response signature and tumor cell death [37, 38].

Second, LINE‐1 transcription or retrotransposition can induce DNA damage. LINE‐1 comprises roughly 500 000 copies in mammals [39, 40], of which only ∼100 remain active in humans [41]. LINE‐1 consists of two ORFs, encoding proteins essential for retrotransposition. ORF1 is an RNA‐binding protein; ORF2 encodes reverse transcriptase and endonuclease activities, crucial for RNA to DNA conversion and integration [42]. Overexpression of LINE‐1 ORF2 can induce DNA damage through its endonuclease activity. For example, the transfection of HeLa cells with LINE‐1 resulted in the formation of γ‐H2AX foci that are evidence of double‐strand breaks (DSBs) [43]. Similarly, in the hematopoietic system, irradiation induces the derepression of LINE‐1 in hematopoietic stem cells (HSCs). Their retrotransposition correlates with the accumulation of DSBs and a loss of HSCs reconstitution capacities. Reverse transcriptase inhibitors suppress these effects [44]. Moreover, it has been shown that LINE‐1 transposition can activate apoptosis through the p53 pathway, as the inactivation of *TP53* reduced apoptosis in a colorectal cancer cell line transfected with LINE‐1 plasmid [45].

Retrotransposon expression and transposition can thus have detrimental effects on cancer cells. By triggering apoptosis or immune response, they will decrease the fitness of their host cells, potentially changing the course of clonal evolution. Cancer cells may avoid such detrimental effects through the overexpression of genes that repress retrotransposons. One example is the overexpression of *SETDB1* in AML. Downregulation of *SETDB1* in AML cell lines resulted in overexpression of retrotransposons and cell death [46]. The depletion of *MPP8*, which is a component of the human silencing hub (HUSH) complex that recruits SETDB1 to chromatin, led to similar results [47].

### 2.2 Retrotransposons Can Increase Cancer Cells’ Fitness

Contrasting the risks of cell death or elimination associated with retrotransposon derepression, retrotransposons can also contribute to oncogenesis. We will distinguish two broad types of contributions that retrotransposons have to clonal evolution: (1) they can contribute to intratumor heterogeneity, and (2) they can indirectly contribute to the process of oncogenesis by modulating oncogenes and tumor suppressor genes.

Through the DNA damage they cause, retrotransposons can contribute to intra‐tumoral heterogeneity [48], provided that their expression does not lead to a viral mimicry response. LINE‐1 insertion can also lead to chromosomal rearrangements [49]. They can also contribute to transcriptomic heterogeneity through the induction of alternative splicing and by acting as secondary promoter to genes [50, 51, 52]. In a multi‐omic analysis of multi‐sampling from autopsies of patients with pancreatic ductal adenocarcinoma, LINE‐1 insertion sites across metastases where found to only partially overlap, showing intra‐patient heterogeneity [53]. Their level of expression may also differ between cancer cells, including between cancer cells with the same genetic background [54], which could also contribute to intra‐clonal heterogeneity. Note that whether such heterogeneity translates into functional heterogeneity and in differential fitness is a question that must be empirically determined.

Retrotransposons derepression can result in ectopic expression of oncogenes and their retrotransposition can disrupt tumor suppressor gene expression [9, 26, 40]. Retrotransposition events are often found in gene sequences, a phenomenon called insertional mutagenesis [9]. Lee et al. observed that more than 40% of identified retrotransposon insertions in five cancer types occurred in tumor suppressor genes [9]. This can hinder their activity. For example, in hepatocellular carcinomas, germline LINE‐1 insertions were found in the *Mutated in Colorectal Cancers* (*MCC*) gene, a tumor suppressor gene that regulates the Wnt/β‐catenin signaling pathway in the liver [55]. Cancer cells harboring LINE‐1 insertion in *MCC* exhibited a drastic reduction of *MCC* expression and showed higher proliferation. ERVs can serve as promoters for transcription through their own promoter contained in their LTR. When inserted near oncogenes, this can trigger their expression and favor cancer cell proliferation. For example, in colorectal cancer, the oncogene *POU5F1B* has been shown to be expressed through the control of an ERV LTR promoter, and to lead to the development of larger tumors in mice [56]. More generally, LINE‐1 is largely expressed in colorectal cancer and has long been thought to contribute to oncogenesis [55]. LINE‐1 knock‐down colorectal cancer cell lines exhibited reduced invasive capacities, and they exhibited reduced cellular migration and tumor growth when injected in mice compared to LINE‐1 derepressed cells [52]. In colon and prostate cancers, De Luca et al. [57] observed that RNA interference or reverse transcriptase inhibitors of LINE‐1 ORF2 impairs the proliferation of cancer cells, indicating that ORF2 protein expression provides them a fitness advantage. Similarly, in acute myeloid leukemia (AML) cell lines, deletion of specific ERV through CRISPR‐Cas9 led to the repression of specific leukemic genes, and the silencing of one ERV family by CRISPR interference suppressed cell proliferation [58].

In summary, retrotransposons can both contribute to intra‐tumor heterogeneity through insertional mutagenesis and induction of alternative splicing, and to oncogenesis through the inactivation of tumor suppressor or over‐expression of oncogenes.

### 2.3 Selection for Optimal Retrotransposon Activity in Cancer?

Two contrasting types of observations have been made. On the one hand, using the Cancer Genome Atlas database, it was observed that retrotransposon expression correlates with type I IFN response expression and estimates of CD8^+^ T cells infiltration [59]. Moreover, in at least some cancers, in particular myeloid malignancies, the level of expression of retrotransposons has been observed to be lower in key cancer cells (leukemic stem cells, which are the main units of selection in cancer [60]) and in more aggressive cancers, indicating negative selection against cancer cells with high retrotransposon activity [61]. On the other hand, it is also now clear that hypomethylation of retrotransposons [28], retrotransposon expression [27] and retrotranspositions [9, 26] are common across cancers. These observations are more challenging to interpret. They could reflect a positive selection of cancer cells with retrotransposon activity, or simply be bystanders of the genetic and epigenetic alterations in cancer cells. As emphasized by the philosopher Elliott Sober, “selection of” does not mean “selection for”: [62] the fact that cancer cells with retrotransposons activity are positively selected (selection *of* cells with such activities) does not mean that they are selected *because* of this retrotransposon activity (selection *for* such activities).

Given that retrotransposon activity partly depends on the epigenetic state of the cells, two scenarios of selection are plausible. The reexpression of retrotransposons in cancer cells could reflect the positive selection of cells with a certain epigenetic state, rather than with a certain retrotransposon activity. This would be a case of selection *of* but not *for* retrotransposons activity. Alternatively, if retrotransposon expression causally increases the fitness of the cells, then it would be a genuine case of selection *for* this activity. The difficulty in interpreting observations comes from the fact that both scenarios can lead to the same outcome: the selection of cells with a certain retrotransposon activity. Some of the examples discussed in Section 1.2 show it is possible to decrease cancer cells proliferation by downregulating LINE‐1 expression. Such mechanistic interventions are in favor of a causal role of LINE‐1 expression in cell fitness, and therefore of a positive selection *for* LINE‐1 expression in these cancer cells.

If retrotransposons expression is subject to both positive and negative selection, it raises the question of whether there is an “optimal” retrotransposon activity—beneficial for positive selection while avoiding negative selection. The question is both conceptually and technically challenging to address because the activity of retrotransposons is plural, and variations can be quantitative (e.g., level of expression) as well as qualitative (e.g., where they retrotranspose, which oncogenes or tumor suppressor genes expression they impact).

There are evidence of a quantitative threshold of retrotransposon expression associated with negative selection. The development of therapeutic strategies aiming to increase retrotransposon expression in order to induce cancer cells death provides experimental illustrations of this quantitative aspect [23, 24, 25, 32, 33]. For example, in chronic myelomonocytic leukemia (CMML), we showed that hypomethylating agents or G9A/GLP H3K9me2 methyltransferase inhibitors (H3K9me2 being histone marks repressing the expression of retrotransposons) alone are not sufficient to trigger retrotransposon overexpression to a level that induces antiviral responses. Their combination is needed to selectively lead to the apoptosis of leukemic cells [25]. However, the level of retrotransposon activity that cells can tolerate may vary, depending on other parameters such as genetic alterations. For example, some cancer cells can resist negative selection due to deleterious effect of retrotransposons when they acquire mutations in downstream pathways, such as TP53 mutations [63]. As a concrete experimental example, Ardeljan et al. showed that the CRISPR knockout of TP53 can partially rescue the reduced clonogenicity observed upon LINE‐1 induction [29].

Data supporting positive selection discussed in Section 1.2 have a more qualitative aspect, showing that retrotransposons may increase cell fitness if they hit specific functional genes—if they insert into tumor suppressor (e.g., the *MCC* gene in hepatocellular carcinomas [55]) or serve as promotors or enhancers of oncogenes (e.g., *POU5F1B* in colorectal cancer [56] or *APOC1* in AML [58]).

Overall, the optimal retrotransposon activity for cancer cell fitness is currently largely unknown and may depend on multiple factors, including cancer types and cell types [64], as well as cancer stage [61], each of which plausibly faces different selective pressure. Progress on this question will be helped by the fast development of CRISPR tools that allow modulation of gene expression. They rely on the use of defective Cas proteins that recruit either activators or inhibitors of transcription [65]. They have already been used successfully to modulate the expression of several members of the same retrotransposon subfamily [47, 58]. The modulation of expression could be fine‐tuned depending on the tool used [66, 67, 68] and may be used to assess the “optimal” level of retrotransposon subfamily activity for different cell types and cancers.

**FIGURE 1:**
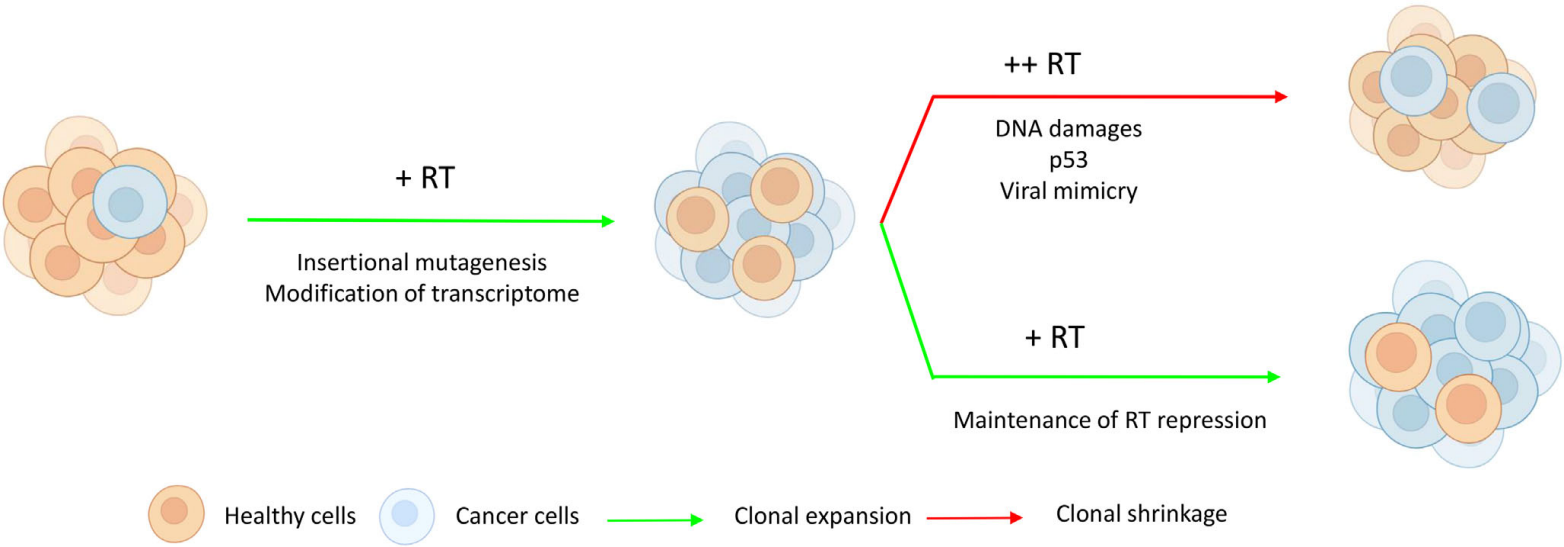
Schematic representation of fitness variation in cancer cells induced by retrotransposons. Increased transcription of retrotransposons and retrotransposition can contribute to oncogenesis and clonal expansion, but a too high increase can lead to clonal shrinkage.

In summary, far from being junk DNA, retrotransposons contribute to clonal evolution in several ways (Figure 1). They can contribute to the diversification of cancer cells, an essential component of clonal evolution. They can also modulate the fitness of the cell: either decreasing it through induction of apoptosis or immune response, or by increasing it through overexpression of oncogenes and immune evasion. This tight relationship with cancer cell fitness makes them an interesting target to modulate clonal evolution. Their contribution to clonal evolution, and the qualitative and quantitative characteristics by which they increase or decrease the fitness of cancer cells, might depend on the cancer types and stages, which should be taken into account when using them as biomarkers [27, 69, 70].

## 3 Retrotransposons May Challenge the Cell as the Sole Unit of Selection in Clonal Evolution

The clonal evolution model is a model of evolution of cancer *cells*. It currently takes the cells as the sole units of selection. However, in principle, any population of entities that exhibit heritable variation in fitness can evolve through natural selection [71, 72]. As such, natural selection can operate at various levels of organization simultaneously, from super‐organismal to sub‐cellular levels. It therefore makes sense to ask whether retrotransposons can also be a unit of selection as they can multiply independently of cell replication, and there is variation among retrotransposons, through the accumulation of mutations. Given the capacity for replication and heritable variation, at the very minimum, retrotransposons could undergo neutral evolution. If the reproductive success of retrotransposons (i.e., how many copies of themselves they insert in the genome) depends on their intrinsic properties (such as their mutations or length), then there would be evolution by natural selection at the level of the retrotransposons. For example, if a mutation decreases or abrogates a retrotransposon's ability to retrotranspose, then it will make less copies of itself, and as a direct consequence, its fitness will be decreased. Several evolutionary biologists have argued that retrotransposons may undergo their own selective pressure [73, 74]. But to our knowledge, evolution of retrotransposons in cancer cells has not yet been studied, and it is therefore unknown if retrotransposons are also a meaningful unit of selection in cancer [75].

Of course, the evolution of retrotransposons and cells are intertwined. The research described in Section 1 illuminates both the beneficial and detrimental roles of retrotransposons on cancer cells’ fitness. While, the multiplication of retrotransposons benefits the retrotransposons themselves, it poses a threat to the fitness of the cancer cell by triggering apoptosis, activating the p53 pathway, and initiating viral mimicry responses and adaptive immunity. In this scenario, selection at the cell level is at least partly dependent on lower‐level selection—the retrotransposon level—and vice versa. Understanding this phenomenon of selection for optimal retrotransposon expression at both the cell and retrotransposon level could provide deeper insight into the fitness of cancer cells (which is what ultimately matters to clonal evolution). However, a full understanding of retrotransposons’ dynamics, especially at the retrotransposon level, may require more efforts to disentangle and clarify retrotransposons’ fitness and more attention paid to genome “ecology,” which, according to Linquist et al., counts as an ecosystem for retrotransposons and is a key determinant to the mobility and functional effect of retrotransposons [74]. In the case of cancers, the impacts of genomic alterations on retrotransposons ecology remains poorly studied, but the epigenetic ecology of retrotransposons is increasingly understood.

Now that the functional role of retrotransposons in cancer is gaining recognition, new questions arise, whose exploration may deepen our understanding of their contribution to cancer biology—such as whether they might represent an underexplored additional unit of selection, and how their ecology within cancer cells could be better understood and perhaps modulated.

## 4 Retrotransposons May Challenge the Traditional View of Cancer Cell Lineages

**FIGURE 2:**
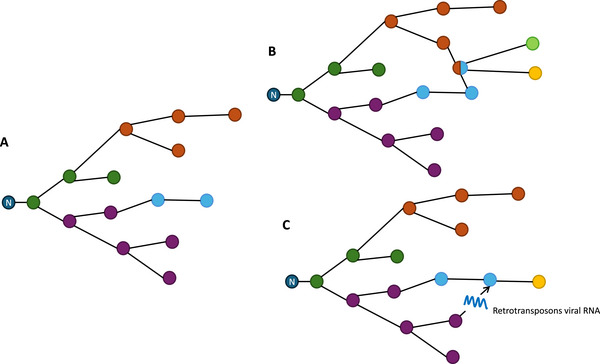
Schematic representations of cancer cell lineages. (A) A traditional tree representation. Lineages are impervious. (B) A tree with cell fusion. (C) A tree with retrotransposons horizontal transfer. *N* = normal cells. Colors represent different genetic clones.

The clonal evolution model relies on a number of assumptions [76], one of which is rarely questioned: that evolution is a branching process only. Unlike sexual reproduction, where each individual inherits properties from two parents, new somatic cells are produced by mitosis from a single parental cell, inheriting properties only from one parental cell. This mode of reproduction is central to the notion of clone: daughter cells are highly similar to the parental cells and to each other. Consequently, the genealogy of cancer cells forms a bifurcating tree through cell division, and new clones emerge when a cell acquires new properties (through one or several co‐occurring alterations), which are then transmitted to descendants. The different clones appearing over time are considered to evolve imperviously as clonal lineages diverge (Figure 2a). In this section, we will explore whether retrotransposons may violate two current assumptions of the clonal evolution model related to the genealogy of cancer cells: (1) that the clonal evolution tree is *only* branching and (2) that branches of the clonal evolution tree are impervious (Figure 2b,c).

### 4.1 Retrotransposon‐Mediated Fusion May Challenge the Branching Process of Clonal Evolution

**FIGURE 3:**
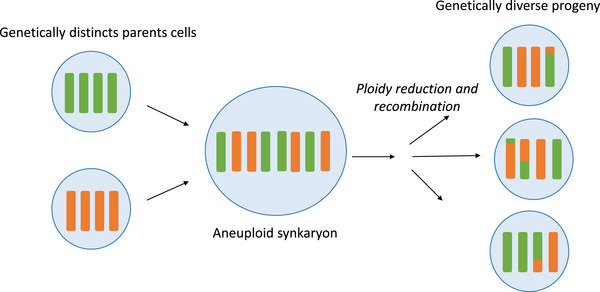
Fused cancer cells can go under ploidy reduction and recombination (inspired by Miroshnychenko et al. [77]). Two parent cells with distinct genetic content can fuse and form an aneuploid synkaryon. This synkaryon can undergo ploidy reduction and recombination to produce a genetically diverse progeny through mitotic division. Such cells contain a mixed genetic content from initial parent cells. Note that fusion can occur between more than two cells.

Although mitosis is the main reproductive process driving clonal evolution, an alternative mode of reproduction, parasexual recombination, can also contribute to clonal evolution through cell fusion [77]. ERVs carry a sequence encoding the fusogenic protein ENV, also called syncytin in mammals, facilitating fusion between the cell and the retrovirus membrane. Although the HERV families that contain the syncytin sequence can no longer retrotranspose, they can still be transcriptionally active. Given the biological function of syncytin, the expression of ERVs in cancers raises the question of whether they could promote the production of new clones through cell fusion (Figures 2b and 3). This alternative clonal evolution model requires three conditions that we will examine in this section: (1) retrotransposons must be able to induce cell fusion; (2) cell fusion must create hybrid cells that are able to divide to give rise to a clone; (3) this clone must be new (i.e., different from the parental clones).

It is well known that retrotransposons can induce cell fusion [78], and several studies have shown that cell fusion can occur in cancers [77, 79, 80, 81]. While most of these studies have not addressed the role of retrotransposons, Strick et al. experimentally demonstrated the involvement of syncytin viral protein in cellular fusion in endometrial carcinoma [81]. They observed a correlation between syncytin overexpression and an increase in cell fusion as well as cell proliferation, and showed that both phenomena were reversed by gene silencing of syncytin. However, their data do not indicate if the fused cells themselves can proliferate.

A study by Marusyk's team, however, provided experimental evidence that hybrids produced from spontaneous fusions of cancer cells can proliferate [77]. They showed that spontaneous cell fusion yields synkaryon, cells containing multiple nuclei which subsequently undergo ploidy reduction and diversification, generating hybrid cells displaying a combination of both parental genotypes (Figure 3). While this study did not investigate the fusion process’ link to retrotransposons, it serves as a proof of principle that fusion per se does not necessarily prevent proliferation. Lastly, several other studies showed that hybrid cells can lead to *new* clones mixing properties from both parental cells [79, 81]. The most detailed investigation to this respect comes from Maruzyk's work, that showed partial recombination, followed by ploidy reduction, which can give rise to not just one but several new clones from one cell fusion event [77].

The possibility that fusion can generate new clones raises questions about the frequency and significance of such events. If fusion does indeed contribute to producing new clones, the central question for the clonal evolution model is whether to include this process or continue to ignore it. As written by the statistician George Box, “All models are wrong, some are useful.” Our goal should be to maximize the usefulness of the clonal evolution model, not focus on its facticity. This prompts two questions: (1) Is there anything problematic in overlooking fusions in the model of clonal evolution? (2) Are there some benefits in including fusion in the model?

While poorly studied, available data suggest cell fusion is a rare phenomenon in tumors [82]. Rarity could be interpreted in two non‐mutually exclusive ways, each with different consequences for the clonal evolution model. It could mean either that few patients or that few cells are affected by cell fusion. If only a small number of patients are concerned, a general clonal evolution model can safely disregard cell fusion, and consider it as an anomaly. Conversely, if fusion is widespread across patients and cancers, its low frequency in individual tumors does not provide a strong argument in favor of ignoring it. Rare events can still have major consequences; in cancer, resistance can come from rare cells, sometimes almost undetectable before treatment [83, 84, 85]. The consequences of resistance, even if rare at the cell level, are unquestionably of central importance for oncology. Thus, the biological and clinical implications of cell fusion might outweigh its low frequency at the cellular level.

Few studies have investigated spontaneous cell fusion in cancers. However, some notable exceptions provide evidence that fusion may be widespread across cancer types and patients. Analysis of tumor biopsies from seven patients with four different solid cancer types revealed gene fusions in all cases [86]. Additionally, several cases of cell fusions between cancer cells and hematopoietic cells in solid tumors have been reported [86, 87]. This suggests that cell fusion might be more widespread among patients than usually considered, though the extent to which it occurs remains an empirical question requiring further investigation.

Concerning the frequency of cell fusion at the cell level, in vivo studies estimated the fusion rate in experimental tumors at 1% [82]. Whereas, a 0.0066% fusion rate was observed in vitro [77]. If fusion occurs at a rate of 1%, a 1 cm^3^ tumor, constituted of approximately 10^9^ cells, may harbor 10^5^ hybrid cells [88]. Those estimates are small but not necessarily negligible, as several studies highlighted functional impacts of cell fusion that are relevant to clonal evolution. Gast et al. showed that transplanted hybrid cells can proliferate faster than the parental cancer cells, suggesting that fusion may increase fitness [86]. The study by Marusyk's team showed that cell fusion could also contribute to the emergence of abnormal chromosomal content favoring variation and adaptation of cancer cells [77]. Hybrids from myeloid and renal carcinoma cell fusion in bone marrow transplanted patients exhibited increased cell movement and secretion of angiogenesis and metastasis promoting factors compared to non‐hybrid renal carcinoma cells [89]. Moreover, polyploid and polynucleated cancer cells attract increasing attention. Like retrotransposons, they were overlooked for a long time. But recent data indicate that they can be involved in resistance to treatment and metastasis [90]. Interestingly, these cancer cells also raise their own set of challenges to the current clonal evolution model, such as the idea that these cells may reproduce without mitosis, through budding, splitting or bursting [91]. While an interesting line of enquiry, exploring this challenge more is beyond the scope of this paper, as the formation of polynucleated giant cells may or may not involve retrotransposons.

Thus, while cell fusion may be rare at the cell level, it may concern a wide range of patients with a wide range of cancers and have relevant impact on the clonal evolution of cancer cells. Fusion can create hybrid cells with unique genetic and functional properties. When able to proliferate, these hybrid cells can contribute to tumor heterogeneity and can lead to the rise of potentially therapy‐resistant clones. Thus, neglecting the role of cell fusion in clonal evolution risks overlooking important causal processes. While most studies on cell fusion do not address how fusions occur, we think that investigating the role of retrotransposons in cell fusion is crucial as their implication may be leveraged in cancer treatment. For example, targeting ASCT1‐2 receptors, the ENV protein, or retrotransposons expression may help avoid retrotransposon‐mediated fusion.

### 4.2 Retrotransposons Horizontal Transfer May Introduce Reticulation in Clonal Evolution

**FIGURE 4:**
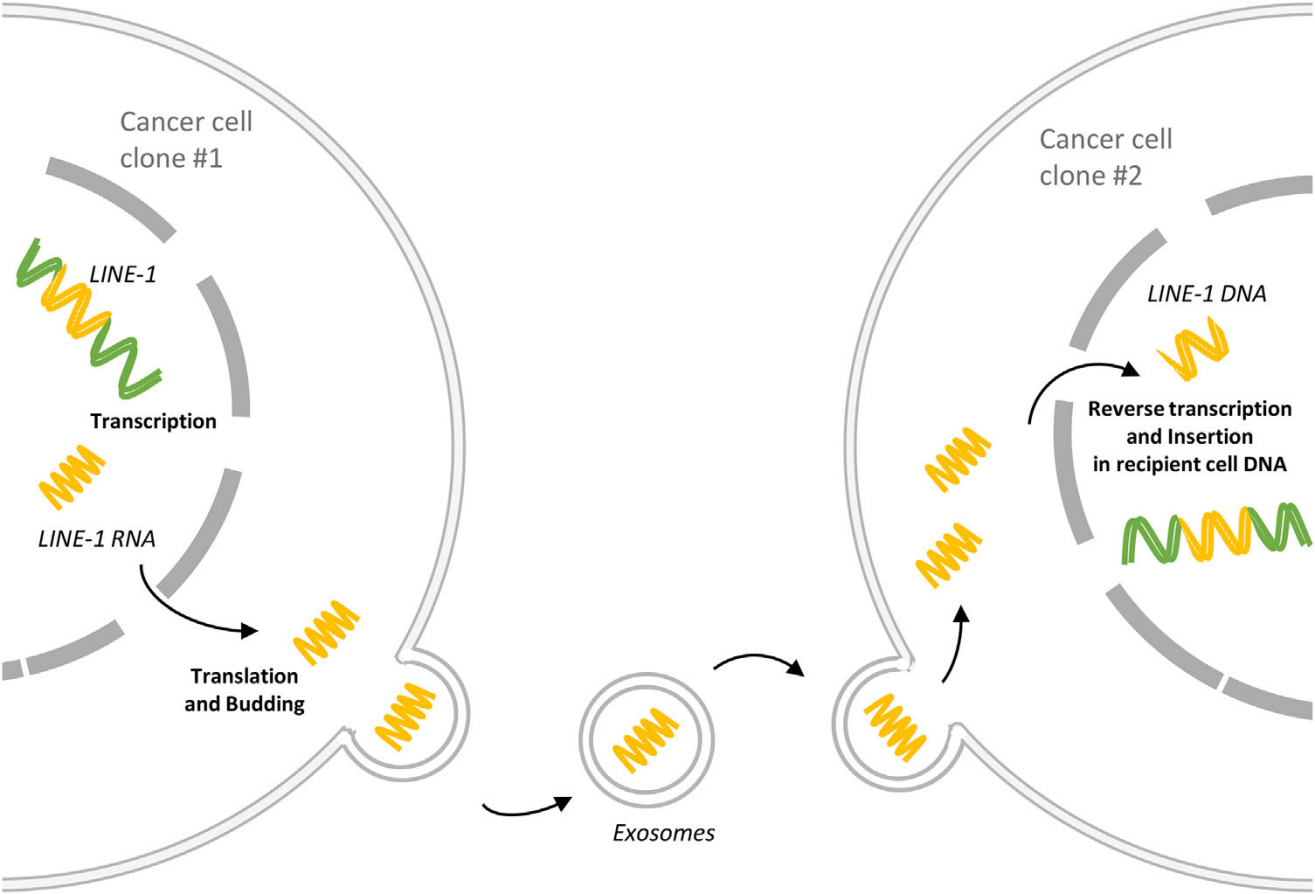
Schematic representation of retrotransposons horizontal transfer in cancer cells. LINE‐1 sequences may be carried out in extracellular vesicles and delivered into another cancer recipient cell, reverse transcribed and integrated in the genome of that recipient cell.

Cancer cell lineages are considered impervious, that is, undergoing their own genetic clonal evolution. Such representation and conceptualization of cancer cell evolution relies on the assumption that cells do not exchange genetic material during evolution. The activity of retrotransposons in cancer cells may question this assumption, as they may introduce horizontal gene transfer (HGT) in clonal evolution. HGT is the movement of genetic material between individuals from different lineages (Figures 2c and 4). It is considered an important source of evolution in the tree of life [92, 93, 94, 95] but it is rarely addressed in cancers. The most acknowledged phenomenon of lateral transfer in cancer is probably the transfer of mitochondria between cells [96, 97, 98]. Some studies have also discussed bacteria‐to‐human lateral gene transfer [99, 100, 101]; others have discussed DNA horizontal transfer through extracellular vesicles (EV) [102], which has been described as a way to transfer drug resistance phenotype to sensitive cancer cells [103]. What about retrotransposons? Several studies have found retrotransposons, especially LINE‐1, in the plasma EVs [69, 104] and tumor derived EVs [105, 106, 107] from different cancers. LINE‐1 could thus also be transferred from one cell to another through EVs. Their uptake can lead to phenotypic changes in the host cell [107, 108]. For example, culture of cancer associated fibroblasts (CAFs) with pancreatic ductal adenocarcinoma‐derived EVs triggered a type‐I IFN response in these cells and a transition from myofibroblastic CAF to inflammatory CAF state [107]. One important question is whether they then integrate into the genome of the host cancer cell, which would ensure not just transient lateral transfer but also the follow up inheritance in the lineage of the recipient cell. Some studies have shown that LINE‐1 in these EVs can be enzymatically active [105, 106], and one study provided an in vitro proof of concept that they can insert in the genome of the host cell [109]. More data is needed to confirm and quantify the occurrence of such transfers and the insertion of LINE‐1 in the host cells.

A more controversial case of lateral transfer of retrotransposons concerns HERV. In humans, HERV are generally older retrotransposons that have lost full replication capacity. However, there has been indirect arguments suggesting that some HERV‐K (the HML2 family) have been active since the divergence between humans and chimpanzees [110]. Moreover, HERV‐K have retained full ORFs [111]. Transcriptional and translational activity has been reported in various cancers, including in teratocarcinoma, melanoma cells lines and patients with essential thrombocytopenia [112, 113, 114]. Virus‐like particles have also been found in the extracellular environment of cancer cell lines and in the plasma of lymphoma and breast cancer patients [115]. Dewannieux et al. also experimentally showed that functional HERV‐K(HML2) can be reconstituted by recombination between genomic loci, questioning the received view that HERV could no longer replicate [116]. Contreras‐Galindo et al. [117] provided evidence that some cancer cell lines can release HERV‐K retrovirus‐like particles that are then taken up by other cells in which they can reverse‐transcribe [117]. Outside of oncology, a recent study has shown that HERV‐K retrovirus‐like particles released by senescent cells can enter non‐senescent cells and induce their senescence [118]. This case is an interesting example of lateral transmission of a phenotype, here mediated by HERV‐K retrovirus‐like particles, which highlights the need for more investigation of whether similar horizontal transfers may happen in cancers, especially in those in which virus‐like particles are found in extracellular environments.

If retrotransposon horizontal transfer does occur (LINE‐1 and/or HERV‐K), then we would have to consider that cancer cell lineages are not as impervious as usually thought and vertical transmission might not be the only form of transmission between cells. This would raise the question of whether the clonal evolution model should incorporate horizontal transfer. As in the case of fusion, existence of lateral transfer would not be sufficient to warrant their integration into the clonal evolution model. This process would need to be widespread across tumors and have functional consequences. One consequence we could anticipate that would warrant revising the assumption that cancer lineages are impervious is if they can create evolutionary short cuts (an adaptive trait is laterally transferred in just one event rather than emerging through a cumulative process of evolution by natural selection over many generations of cells). The impacts of retrotransposons lateral transfer could go both ways: suddenly increasing or decreasing the fitness of a cell and its descendants. A broader question for the field is then whether retrotransposons can influence the pace of evolution.

In summary, retrotransposons may have a broader range of impacts on cancer evolution than we currently think. Recent data raise the question of whether they may interfere with cell lineages by inducing cell fusion or through their horizontal transfer from one cell to another (Figures 2 and 4). Though the current evidence is fragmentary, it invites to further exploration. Taking such forward‐looking perspective, we outlined two types of conditions that would need to be met for retrotransposons to challenge the clonal evolution model: material conditions—their actual implication in cell fusion and their integration into the host genome after lateral transfer—and theoretical conditions—these processes must have functional consequences and be recurrent across tumors.

## 5 Conclusions

More work is being done to fill certain gaps in and improve the clonal evolution model (e.g., [14, 15, 16, 17, 18, 75]). In joining these efforts, we believe it is time to account for the role of retrotransposons in clonal evolution. There is now growing evidence linking retrotransposons to cancer initiation and progression. In this article, we confronted the clonal evolution model with the current knowledge on retrotransposons in cancer.

The primary impact of retrotransposons on clonal evolution is their capacity to modulate fitness. Depending on their context, level of expression and insertion loci, retrotransposons can either positively or negatively impact the fitness of the cancer cell. Understanding their role holds promise for better understanding the dynamics of cancer (sub)clones. The modulation of cancer cell fitness by retrotransposons is compatible with the current model of clonal evolution. Its integration in the model may require adopting a multi‐level selection perspective. Such integration would enrich, rather than challenge, the clonal evolution model. Furthermore, as drugs affecting DNA methylation and chromatin modifications can modulate retrotransposon expression, they offer the potential for therapeutic strategies for controlling clonal expansion or eradicating some clones.

Another more speculative impact of retrotransposons on clonal evolution is their potential interference with cell lineages, which may challenge some of the current assumptions hold by the clonal evolution model. As somatic cells reproduce by mitosis, their lineages are considered as bifurcating trees, with vertical inheritance of the genome from the parent cells to their daughter cells. This vision of lineages, and the associated idea that lineages are impervious have been questioned and criticized for being too simplistic [119, 120, 121]. Here we discuss the possibility that retrotransposons also contribute to lineage violations as they can induce cell‐cell fusion, altering the inheritance pattern within cancer cell lineages by mixing genomes from two parent cells. Although evidence suggests retrotransposons can induce fusion, and cell fusion can confer evolutionary advantages to hybrid clones, the relationship between the two requires further investigation. Lastly, retrotransposons can transfer between cancer cells, in particular through EV, potentially introducing horizontal inheritance alongside vertical inheritance in cancer cell lineages. While formal evidence of this process is limited, we argue that the plausibility and potential impacts of such lineage violations warrant further investigation.

## Author Contributions

A.L. did the literature research and analysis, and wrote the first version of the paper, on the supervision of L.L. F.P., E.E.M., and L.L. contributed to the rewriting of the paper.

## Data Availability

Data sharing is not applicable to this article as no new data were created or analyzed in this study.
